# Current Therapeutic Strategies for Glioblastoma

**DOI:** 10.3390/brainsci10010015

**Published:** 2019-12-26

**Authors:** Julien Rossignol, Bhairavi Srinageshwar, Gary L. Dunbar

**Affiliations:** 1 Field Neurosciences Institute laboratory for Restorative Neurology at Central Michigan University, Mt. Pleasant, MI 48859, USA; srina1b@cmich.edu (B.S.);; 2 Program in Neuroscience, Central Michigan University, Mt. Pleasant, MI 48859, USA; 3 College of Medicine, Central Michigan University, Mt. Pleasant, MI 48859, USA; 4 Department of Psychology, Central Michigan University, Mt. Pleasant, MI 48859, USA; 5 Field Neurosciences Institute, St. Mary’s of Michigan, Saginaw, MI 48604, USA

**Keywords:** glioblastoma, drugs and genes, blood brain barrier

## Abstract

Glioblastomas (GB) are grade 4 brain tumors, one of the most aggressive forms of brain cancer found in humans. Although current treatments for GB are largely ineffective, new alternate approaches, beyond standard chemo- or radiation therapies have shown promising results in both pre-clinical and clinical settings. Some of these approaches include stem cell therapy, new pharmaceuticals and nutraceuticals, and the use of tumor specific peptides or GB-targeted antibodies and immune-based therapies. A common limitation in the efficacy of these treatments is the inability of these therapeutic agents to readily cross the blood–brain barrier (BBB) to reach the tumor site. Therefore, many strategies are being developed to achieve targeted delivery of drugs across the BBB so that they can kill the cancer cells, while sparing healthy tissue. One of the most promising new approaches involves the use of nanoparticles that can carry therapeutic drugs and genes across the BBB and home in on the GB tumor site.

Under the broad category of different forms of tumors that arise from glial cells, the most aggressive form is the glioblastoma (GB), a grade-4 astrocytoma. There is no cure or effective treatment for GB and patients with this deadly form of cancer have a high rate of mortality [1], usually within 15–18 months of diagnosis. Current strategies for treating GB involve: (1) chemotherapy and radiation therapy; (2) surgical removal of tumor; and (3) use of drugs and therapeutic genes that target the tumor. All of these strategies have their own advantages and disadvantages depending on how well the tumor site can be targeted and destroyed without damaging the healthy tissue surrounding the tumor. The side effects of radiation therapy often include aberrant immunological effects and cytotoxicity [2]. In addition, because GB cells are highly heterogeneous in nature, there is significant variability in how the cells respond to radiation, limiting its effectiveness to a small sub-population of the GB cells. Moreover, the cancer stem cells (CSC) that are present at the center of the tumor are often difficult to target and give rise to more aggressive forms of tumor regrowth following radiation. An alternate approach is to surgically remove the tumor(s), but this procedure is fraught with the serious limitation, in that primary GB tumors diffuse throughout neighboring tissue and are seldom localized to a single region [3,4].

However, there are many alternative options available for managing and treating GB tumors. An interesting up-to-date concise review article, “Glioblastoma under Siege: An Overview of Current Therapeutic Strategies” published in *Brain Science* outlines different types of treatment strategies, including gene therapy, immunotherapy, and the use of drugs for treating GB. The authors discuss the genetic and the epigenetic changes that occur in the GB brain following tumor formation, including upregulation and downregulation of various factors and genes, which, in turn, affect certain pathways leading to greater proliferation and invasion of the tumor. For example, mutations found in phosphate and tensin homologue (*PTEN*) and epidermal growth factor receptor (*EGFR*) genes are found in primary forms of GB. In addition, cell cycle/check point related mutations (p53) and chromosomal loss are associated with secondary forms of the tumor. Moreover, there are several epigenetic alterations found in the GB brain, such as deletion of micro RNAs (e.g., miR4484) that are important for tumor suppression, which lead to altered transcription and translation of genes that enhance tumor growth. Therefore, restoring regulation of the genetic and epigenetic markers in the GB brain promises to be a viable treatment option. 

Other treatment options described in the review include the use of drugs (either individually or in combination) that interfere with the tumor cell cycle to halt tumor growth. One current combinatorial treatment includes radiation along with temozolomide (TMZ) to arrest the cell cycle at the G2/M phase, causing cell death in the tumor. However, the effects of TMZ (which acts by methylating DNA at specific sites) can be undone by DNA repair mechanisms, especially in the presence of the enzyme methylguanine methyltransferase (MGMT). Therefore, to avoid this, a combination of TMZ, along with capecitabine, was developed, in which the capecitabine inhibits MGMT, thereby suppressing DNA repair mechanisms in the tumor. Another option, immune-based therapy, relies on specific monoclonal antibodies that target specific surface receptors (such as EGFRvIII and VEGF) on the tumor. However, this approach was ineffective, relative to placebos, in phase II clinical trials. Nonetheless, complexing these antibodies with toxic molecules or drugs to target and specifically kill the cancer cells remains a promising strategy for treating GB. The antibody-drug conjugate, along with TMZ, are currently in phase I and II clinical trials and have shown some promising results. Other approaches involve the use of oncolytic viruses, such as HSV1, that can be targeted to tumors (based on cell-specific mutations), a new strategy that is currently being tested in clinical trials [5]. 

Another recent development is the use of recombinant poliovirus (polio–rhinovirus chimera; PVSRIPO) to kill tumors, a strategy that was very successful and is currently in phase II clinical trials. The virus was injected intracranially into the brain of GB patients and the study outcome showed that overall patients survived for longer, much more than the average 18-month lifespan of a GB patient. While patients in the control group tended to decline quickly, the patients who received the virus remained stable 24 months after treatment [6]. 

Having discussed the different types of genetic, epigenetic, drug, and immune based therapies available, there are important factors that need to be addressed for successful targeted delivery of potential treatments, particularly systemic delivery across the BBB. Most of the current treatments do not cross the BBB and need to be delivered via direct intracranial injections, making the use of nanoparticles that can carry drugs and biomolecules across the BBB a particularly attractive alternative. We and others have used specific nanoparticles, known as dendrimers, to carry and deliver genes and drugs for treating deficits in animal models of neurodegenerative diseases and GB. In addition, current methods of delivering genes employing viral vectors, such as adeno-associated virus (AAV9) carrying interferon β (IFNβ), when injected systemically into GB mice can cross the BBB and reach the tumor site and cause complete regression of the tumor [7]. However, AAVs have limited cargo-carrying capacity making it is nearly impossible to deliver multiple genes to cells. As an alternative, nanoparticles, specifically dendrimers, offer a viable option, as they can form complexes with large constructs containing multiple genes and deliver them to cells *in vitro* and *in vivo*. Our dendrimers, specifically, are surface-modified to reduce the cytotoxicity that has been observed in commonly used amine surface dendrimers [8,9], and these specially formulated dendrimers can carry a relatively large cargo and deliver it safely across the BBB.

In conclusion, although GB has proved to be refractory to most therapeutic strategies, there are several new approaches, which include targeted delivery to the tumor in a safe and effective manner. Given this, there is reason to be optimistic that new, effective treatments for GB that meet clinical standards will be developed in the near future.
